# Dichloroacetate restores drug sensitivity in paclitaxel-resistant cells by inducing citric acid accumulation

**DOI:** 10.1186/s12943-015-0331-3

**Published:** 2015-03-19

**Authors:** Xiang Zhou, Ruohua Chen, Zhenhai Yu, Rui Li, Jiajin Li, Xiaoping Zhao, Shaoli Song, Jianjun Liu, Gang Huang

**Affiliations:** Department of Nuclear Medicine, Ren Ji Hospital, School of Medicine, Shanghai Jiao Tong University, 1630 Dongfang Road, Shanghai, 200127 China; School of biomedical engineering, Shanghai Jiao Tong University, Shanghai, China; Department of Cancer Metabolism, Institute of Health Sciences, Chinese Academy of Sciences and Shanghai Jiao Tong University School Medicine, Shanghai, China

**Keywords:** Dichloroacetate, Paclitaxel, Mitochondrial respiratory defect, Pyruvate dehydrogenase kinase, Warburg effect

## Abstract

**Background:**

The Warburg effect describes the increased reliance of tumor cells on glycolysis for ATP generation. Mitochondrial respiratory defect is thought to be an important factor leading to the Warburg effect in some types of tumor cells. Consequently, there is growing interest in developing anti-cancer drugs that target mitochondria. One example is dichloroacetate (DCA) that stimulates mitochondria through inhibition of pyruvate dehydrogenase kinase.

**Methods:**

We investigated the anti-cancer activity of DCA using biochemical and isotopic tracing methods.

**Results:**

We observed that paclitaxel-resistant cells contained decreased levels of citric acid and sustained mitochondrial respiratory defect. DCA specifically acted on cells with mitochondrial respiratory defect to reverse paclitaxel resistance. DCA could not effectively activate oxidative respiration in drug-resistant cells, but induced higher levels of citrate accumulation, which led to inhibition of glycolysis and inactivation of P-glycoprotein.

**Conclusions:**

The abilityof DCA to target cells with mitochondrial respiratory defect and restore paclitaxel sensitivity by inducing citrate accumulation supports further preclinical development.

**Electronic supplementary material:**

The online version of this article (doi:10.1186/s12943-015-0331-3) contains supplementary material, which is available to authorized users.

## Background

More than 80 years ago, Warburg first observed that glycolysis is increased in cancer cells that depend largely on this metabolic pathway to meet their energy needs [1]. Positron emission tomography (PET) has since confirmed that most tumors undergo increased glucose uptake and metabolism [2]. This phenomenon is known as the Warburg effect, which is believed to reflect mitochondrial injury and alternative isoforms of glycolytic enzymes in cancer cells [3,4]. Mitochondrial respiratory injury is due primarily to mutations in mitochondrial DNA, reduced activity of key tricarboxylic acid (TCA) cycle enzymes, and a hypoxic microenvironment. Tumor cells with mitochondrial respiratory injury have decreased TCA cycle and/or ETC. flux, which can lead to reduced production of CO_2_ and ATP with decreased O_2_ consumption [5-7]. As a result, targeting mitochondria in cancer therapy is receiving increased attention.

Dichloroacetate (DCA) activates pyruvate dehydrogenase complex (PDC) by inhibiting pyruvate dehydrogenase kinase (PDK). PDC is an important gateway enzyme that influences glycolysis and glucose oxidation [8]. Activation of PDC converts pyruvate into acetyl-CoA, which is converted into citric acid in the mitochondria, stimulating mitochondrial oxidative phosphorylation [9].

DCA induces apoptosis, decreases proliferation, and inhibits tumor growth through inhibition of PDK [10]. DCA has no apparent toxicity except for peripheral neuropathy, which is reversible upon drug discontinuation [11]. Recently, DCA has been used increasingly in research on prostate, breast and colorectal cancer [12-14]. DCA toxicity is specific to the tumor type [15-17], and tumor cells with mitochondrial injury are sensitive to DCA through an unknown mechanism [18].

Paclitaxel is an important clinical agent for the treatment of malignant breast, prostate and non-small lung tumors, but resistance is a serious problem [19,20]. Contributing to drug resistance is the high expression of anti-apoptotic proteins as well as the high expression of proteins that lets cancer evade cancer treatment [21], and finding a way to reverse multidrug resistance is a major target of cancer research.

A549/Taxol cells are paclitaxel resistant and exhibit high levels of P-glycoprotein expression [22]. In this study, we observed that DCA targets A549/Taxol cells to enhance the sensitivity to paclitaxel. Given that DCA is known to target cells with mitochondrial respiratory defects [18], we investigated whether the mitochondria of A549/Taxol cells were damaged. DCA activates PDC to stimulate mitochondria, therefore we assessed whether DCA specifically targets drug-resistant cells because of its restoration of mitochondrial function.

We observed that the paclitaxel resistance in lung cancer cells was closely correlated with mitochondrial injury. DCA could not activate oxidative respiration in drug-resistant cells but did inhibit glucose uptake significantly. These results have improved our understanding of the mechanism by which DCA affects tumor cell suppression, mitochondrial function, glucose metabolism and drug resistance.

## Materials and methods

### Chemicals and reagents

DCA, 3-bromopyruvate, sodium pyruvate, coenzyme A, glutamine, uridine and daunomycin were purchased from Sigma. Dulbecco’s Modified Eagle’s Medium (DMEM) and fetal bovine serum (FBS) were purchased from GIBCO. ^14^C-6-glucose was purchased from Moravek Biochemicals. Mouse monoclonal anti-P-glycoprotein was purchased from Santa Cruz Biotechnology and rabbit polyclonal anti-PDK2 was purchased from Protein Tech. ^99m^Tc-MIBI (methoxyisobutylisonitrile) was purchased from Shanghai Hinko and ^18^ F-fluorodeoxyglucose was produced in our laboratory.

### Cell culture and transient transfection

The human lung adenocarcinoma cell lines A549and H1299wereobtained from the Chinese Academy of Sciences. HCCLM3 cell line [23] (human HCC cell lines with high metastatic potentials established at the Liver Cancer Institute, Zhong Shan Hospital, Fudan University, China) was also used in this study. The paclitaxel-resistant A549/Taxol cell line was developed as previously described [22,24]. Briefly, the parental A549 cell line was exposed to an initial paclitaxel concentration of1 μg/L. The concentration of paclitaxel was escalated stepwise to 1 μg/mL with changes of medium biweekly. The surviving resistant cells were renamed A549/Taxol and cultured in DMEM containing 5 mM glucose supplemented with 10% FBS, sodium pyruvate and l-glutamine. The mitochondrially defective clone, A549/MD cells, was produced as previously described [25]. Briefly, the cells were produced by exposure to 5 ng/ml ethidium bromide, which was increased in a stepwise fashion to a limit of 50 ng/ml. A549/MD cells were maintained in DMEM supplemented with 10% FBS, 1 mM sodium pyruvate, 50 mM uridine, 5 mM glucose and 2 mM l-glutamine. Hypoxic treatment was performed in a specially designed hypoxia incubator (Chermo Electron) with 1.5% O_2_, 5% CO_2_ and 93.5% N_2_. Lipofectamine 2000 (Invitrogen) was used for transient transfection. siRNA-PDK2 and control was purchased from Pharma China.

### Measurement of intracellular ATP

Relative cellular ATP content was measured using the ATP bioluminescent somatic cell assay kit (Sigma). Cell lysates were collected, and luminescence was measured using a luminescence reader and normalized to the protein concentration.

### Mitochondrial membrane potential

Cells were plated insix-well plates with 2 × 10^5^cells per well. 5,5′,6,6′-tetrachloro-1,1′,3,3′-tetraethylbenzimidazol-carbocyanineiodide (JC-1) was added to the medium at a final concentration of 10 μg/ml, and the cells were allowed to stain for30 min. After JC-1 staining, cells were washed twice with PBS and resuspended in 300 μl PBS. JC-1 intensity was examined using fluorescence-activated cell sorting. Mitochondrial membrane potential was calculated using the followingformula:JC-1 intensity of Q2 quadrant/JC-1 intensity of Q4 quadrant.

### Analysis of enzyme activity

For analysis of complex I activity from A549, A549/Taxol and A549/MD cells, the Complex I Enzyme Activity Microplate Assay Kit was used (Abcam). Cell lysates were collected and added to each well of the microplate and incubated for 3 h. After rinsing all wells, assay solution was added and absorbance at 450 nm was measured at ~1-min intervals for 30 min.

Phosphofructokinase (PFK) activity was measured using the PFK activity colorimetric assay kit (BioVision). A549, A549/Taxol cells were treated with DCA for 24 h. Cell extracts were prepared and assayed and absorbance at 450 nm was measured at 10, 20 and 30 min.

For pyruvate dehydrogenase (PDH) activity measurement, mitochondria from A549 and A549/Taxol cells treated with DCA for 24 h were isolated using the Mitochondria Isolation Kit for Mammalian Cell (Thermo). After mitochondria were sonicated, PDH activity was assayed by measuring the reduction of NAD^+^ at 340 nm in the presence of 200 μM TPP, 40 μM coenzyme A, and 4 μM pyruvate. The assay was carried out in the presence of 2.5 μM rotenone to prevent NADH consumption by complex I [26].

### Oxygen consumption and extracellular acidification rate analysis

Oxygen consumption rate (OCR) and extracellular acidification rate (ECAR) were measured using the Seahorse XF24 analyzer (Seahorse Bioscience). Cells were seeded in a 24-well cell culture microplate and allowed to attach overnight. Approximately 30 min prior to the assay, culture medium was changed to the Seahorse assay medium, DMEM containing 5 mM glucose or 2 mM l-glutamine. The waveguides deliver light rays at various excitation wave lengths (oxygen = 532 nm, pH = 470 nm) and transmit a fluorescent signal (oxygen = 650 nm, pH = 530 nm). OCR and ECAR were measured, as described previously [27,28].

### Metabolite and citrate extraction using GC/MS analysis

Metabolite and citrate extraction was performed using GC/MS analysis, as described previously [29]. Briefly, cells were cultured in 6-cm dishes, rinsed with 1 ml ice-cold PBS, quenched with 1 ml 50% ice-cold methanol, and the extracts were vortexed at 4°C for 30 min. After samples were centrifuged and evaporated, *tert*-butyldimethylsilylderivatization was initiated, and the samples were incubated at 75°C for 1 h. GC/MS analysis was performed using the Agilent 6890GC.

### ^18^ F-FDG uptake

Cells were cultured in 12-well plates, detached, washed twice, and incubated in 500 μl DMEM containing 4 μCi/ml ^18^ F-FDG for 1 h at 37°C. Pellets were washed twice with ice-cold PBS. Lysates were produced using 500 μl 0.1 M NaOH and the radioactivity of the whole-cell lysates was assayed using a well gamma-counter [4]. Cell protein content was measured in parallel using the BCA protein assay kit (BIB).

### ^14^CO2 release assay

Cells cultured in 100-mm dishes were placed in an airtight chamber. Five milliliters of DMEM containing ^14^C-6-glucose (2 μCi/ml) was added and the cells were incubated at 37°C for 2 h with a constant airflow (5% CO_2_ – 95% air, 15 ml/min); ^14^CO_2_ was trapped using 16 ml amine-based absorber. Four milliliters of absorber-trapped ^14^CO_2_ was transferred to vials containing 12 ml permafluor E+ and radioactivity was counted using a liquid scintillation counter [30]. The cell residue was used to determine protein content.

### Analysis of P-glycoprotein activity

^99m^Tc-methoxyisobutylisonitrile (^99m^Tc-MIBI) is a transport substrate for P-glycoprotein [31]. For quantitative assessment of transporter function in multidrug-resistant cells, ^99m^TC-MIBI was added to cultured tumor cells. Uptake and efflux were determined as described previously [32]. Briefly, the cells were cultured in 12-well plates, and the medium was removed and replaced with 500 μl medium containing 4 μCi/ml ^99m^TC-MIBI and various modulators for 30 or 120 min at37°C. The cells were subsequently lysed in 0.1 mM NaOH. Efflux of ^99m^TC-MIBI was performed as described previously [26]. Cells were incubated in DMEM containing ^99m^TC-MIBI and various modulators for 1 h, washed twice with PBS, and the medium without ^99m^TC-MIBI was replaced. The cells were lysed for 10, 30, 60 or 90 min with NaOH. All samples were measured using a well gammacounter. In parallel, cell protein content was measured using the BCA protein assay kit.

Activity of P-glycoprotein was measured by analysis of the intracellular accumulation of doxorubicin, as described previously [33]. The cells were cultured in 12-well plates, containing 10 mg/L doxorubicin and various modulators for 2 h at 37°C. Cells were resuspended in 0.2 ml PBS and analyzed by flow cytometry with an excitation wavelength of 488 nm for the mean fluorescence intensity of intracellular doxorubicin.

### Cell Counting Kit (CCK)-8 assay

Cell survival was measured using the CCK-8 assay (Dojindo Molecular Technologies), as described previously [34]. Cells were plated in 96-well plates at 6000 cells/well. After 24 h culture, the medium was replaced with DMEM containing 10% FBS and drugs at the indicated concentrations. After incubation for 24 h, 10 μlcholecystokinin-8 was added to each well, and the cells were incubated for a further 2 h. Absorbance was read at 450 nm using an enzyme microplate reader. The half-maximal inhibitory concentration (IC_50_) was calculated using GraphPad Prism version 5.0. Resistance index was calculated using the following formula: IC_50_ resistant cells/IC_50_ parental cells.

### *In vivo* effect of DCA and paclitaxel in A549/Taxol cells xenograft

All animal experiments were performed in accordance with the National Institutes of Health Guide for the Care and Use of Laboratory Animals and were approved by Affiliated RenJi Hospital of Shanghai Jiaotong University. Male 4–6-week-old BALB/c athymic (nut/nut) mice (SLAC Laboratory Animals) were subcutaneously inoculated with 5 × 10^6^ A549/Taxol cells in serum-free medium. Mice were randomized into four groups of six 7 days after inoculation: (1) vehicle (control); (2) paclitaxel alone; (3) DCA alone; and (4) DCA combined with paclitaxel. DCA (0.75 g/L) was added to drinking water for mice in the DCA alone and DCA + paclitaxel groups. Mice in the paclitaxel alone and DCA+ paclitaxel groups were intraperitoneally injected with 6 mg/kg paclitaxel, which was repeated once weekly for a total of three doses (18 mg/kg). Tumor volume was calculated using the following formula: volume (mm^3^) = (width)^2^ × length × 0.5. Tumor volume and body weight were measured twice weekly. Five weeks after treatment, mice were sacrificed and weighed, and tumors were excised and weighed.

### Statistical analysis

Statistical differences between the groups were assessed using two-tailed analysis of variance and *t* tests. *P* < 0.05 was considered statistically significant.

## Results

### Paclitaxel-resistant cells are associated with mitochondrial dysfunction

**Figure 1:**
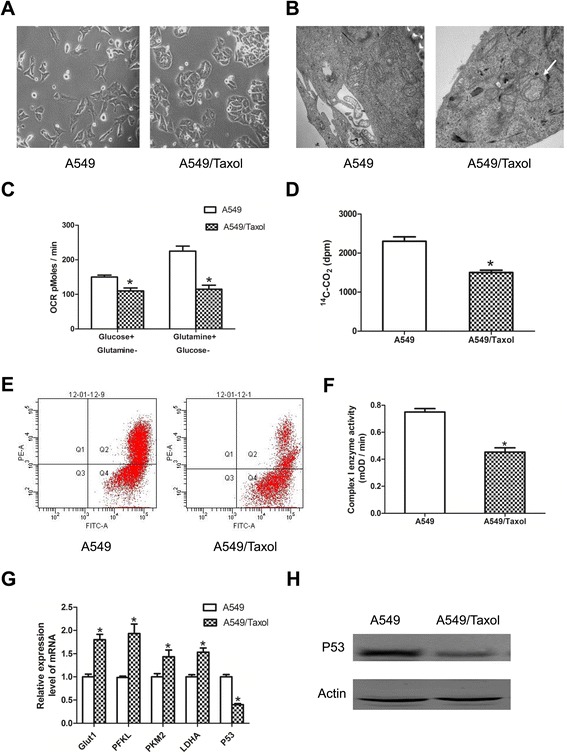
**Characterization of alterations in mitochondrial respiration in paclitaxel-sensitive (A549) and paclitaxel-resistant (A549/Taxol) cells. (A)** A549 and A549/Taxol cells at 200× magnification. **(B)** Transmission electron microscopy of mitochondrial morphology. **(C)** Oxygen consumption. Cells were cultured in DMEM using glucose or glutamine as a carbon source. **(D)** Release of ^14^C-CO_2_. Cells (5 × 10^6^) were cultured in 4 ml low-glucose DMEM containing 0.5 μCi/ml ^14^C-6-glucose for 2 h. Released ^14^C-CO_2_ was measured. **(E)** Mitochondrial membrane potential was calculated using the following formula: JC-1 intensity of Q2 quadrant / JC-1 intensity of Q4 quadrant. **(F)** Mitochondrial oxidative phosphorylation complex I enzyme activity. **(G)** Comparative analysis of the expression of key glycolysis enzymes. **(H)** p53expression in A549 and A549/Taxol cells by western blot analysis. *P < 0.05. Data are mean ± standard error of the mean (SEM) of three independent experiments.

A549/Taxol cells had a paclitaxel resistance index of 32 (Figure 1A). Paclitaxel increases production of reactive oxygen species (ROS) in tumor cells that may result in mitochondrial injury because of long-term ROS-induced oxidative stress and a compromised mtRNA self-repair mechanism [35].

Electron microscopy showed that mitochondrial volume increased and cristae appeared less wellformed in A549/Taxol cells compared withA549 cells (Figure 1B). Mitochondrial respiratory injury can be evaluated through the oxygen consumption rate (OCR) and release of ^14^C-CO_2_. The oxygen consumption rate of the two cell lines was measured with glucose or glutamine (Figure 1C), and A549/Taxol cells showed significantly reduced oxidative capacity with either carbon source. Release of ^14^C-CO_2_ was measured to evaluate mitochondrial respiration using ^14^C-6-glucose as a carbon source, and A549/Taxol cells released less ^14^C-CO_2_ than A549 cells did (Figure 1D). Mitochondrial membrane potential is an indicator of mitochondrial function in stable cell lines, and damage to the electron transport chain can decrease mitochondrial membrane potential [36]. Lower mitochondrial membrane potential was observed in A549/Taxol cells (Figure 1E), and the activity of complex I was also greatly reduced in these cells compared withA549 cells (Figure 1F). These results demonstrated that the mitochondria of paclitaxel-resistant cells were severely damaged.

Mitochondrial damage in tumor cells can lead to metabolic changes. Analysis of RT-PCR data confirmed that the mRNA levels of key enzymes involved in glucose metabolism were increased, and expression of the tumor suppressor gene p53 was decreased in A549/Taxol compared withA549 cells (Figure 1G). Similarly, the protein expression of p53 was decreased in A549/Taxol cells compared withA549 cells (Figure 1H).

### DCA specifically reverses drug resistance in cells with mitochondrial respiratory deficiency

**Figure 2:**
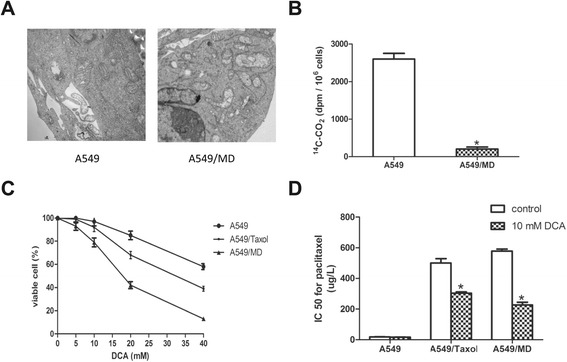
**DCA affects paclitaxel resistance in cells with damaged mitochondria. (A)** Transmission electron microscopy of mitochondrial morphology in mitochondrial respiration-deficient A549/MD cells. **(B)** Release of ^14^C-CO_2_ from A549/MD cells. **(C)** Viability of A549, A549/Taxol and A549/MD cells treated with DCA for 48 h. **(D)** IC_50_ for paclitaxel in A549, A549/Taxol and A549/MD cells after treatment for 48 h with 0 and 10 mM DCA. *P < 0.05. Data are mean ± SEM of three independent experiments.

Mitochondria are pivotal in energy metabolism and regulation of apoptosis [37], therefore we hypothesized that the artificial/induced mitochondrial respiratory defect in A549 cells may increase paclitaxel resistance. A549/MD cells derived from A549 cells were defective in mitochondrial respiration, and transmission electron microscopy revealed that mitochondria in these cells were abnormally swollen with disorganized cristae (Figure 2A). Biochemical analysis showed that A549/MD cells released less ^14^C-CO_2_and had lower minimal complex I activity than A549 cells had (Figure 2B; Additional file 1: Figure S1), indicating a severe deficiency in mitochondrial respiration. The effect of mitochondrial respiratory defects on the cellular response to anti-cancer agents was investigated, and as expected, A549/MD cells possessed a higher resistance to paclitaxel than A549 cells (Figure 2D) with a resistance index of 37.

P-glycoprotein is an important resistance-associated protein that can secrete antitumor drugs, and paclitaxel is a known P-glycoprotein substrate [36]. Expression of P-glycoprotein was increased in the stable A549/MD cell line (Additional file 2: Figure S2), suggesting that it contributes to or is responsible for paclitaxel resistance in A549/MD cells.

Drug resistance profoundly affects the clinical treatment of tumors. Paclitaxel-resistant cells were associated with mitochondrial respiratory defects, therefore preventing drug resistance by targeting mitochondrial respiratory defects and restoring drug sensitivity may be a promising area of research.

DCA is a known regulator of PDK and is thought to act specifically on cells with mitochondrial respiratory defects. In this study, DCA treatment for 48 h resulted in more A549/MD and A549/Taxol cell death with injured mitochondria than occurred in A549 cells (Figure 2C). In addition, DCA significantly increased the sensitivity of drug-resistant cell lines (both A549/Taxol and A549/MD cells) to paclitaxel (Figure 2D), while the parental A549 cells were unaffected. These results indicate a link between drug resistance and mitochondrial respiratory defects, and reversal of drug resistance by DCA is specific to drug-resistant cells exhibiting mitochondrial respiratory defects.

### DCA cannot effectively restore mitochondrial respiration in paclitaxel-resistant cells, but inhibits glycolysis significantly more than A549 cells with functioning mitochondria

**Figure 3:**
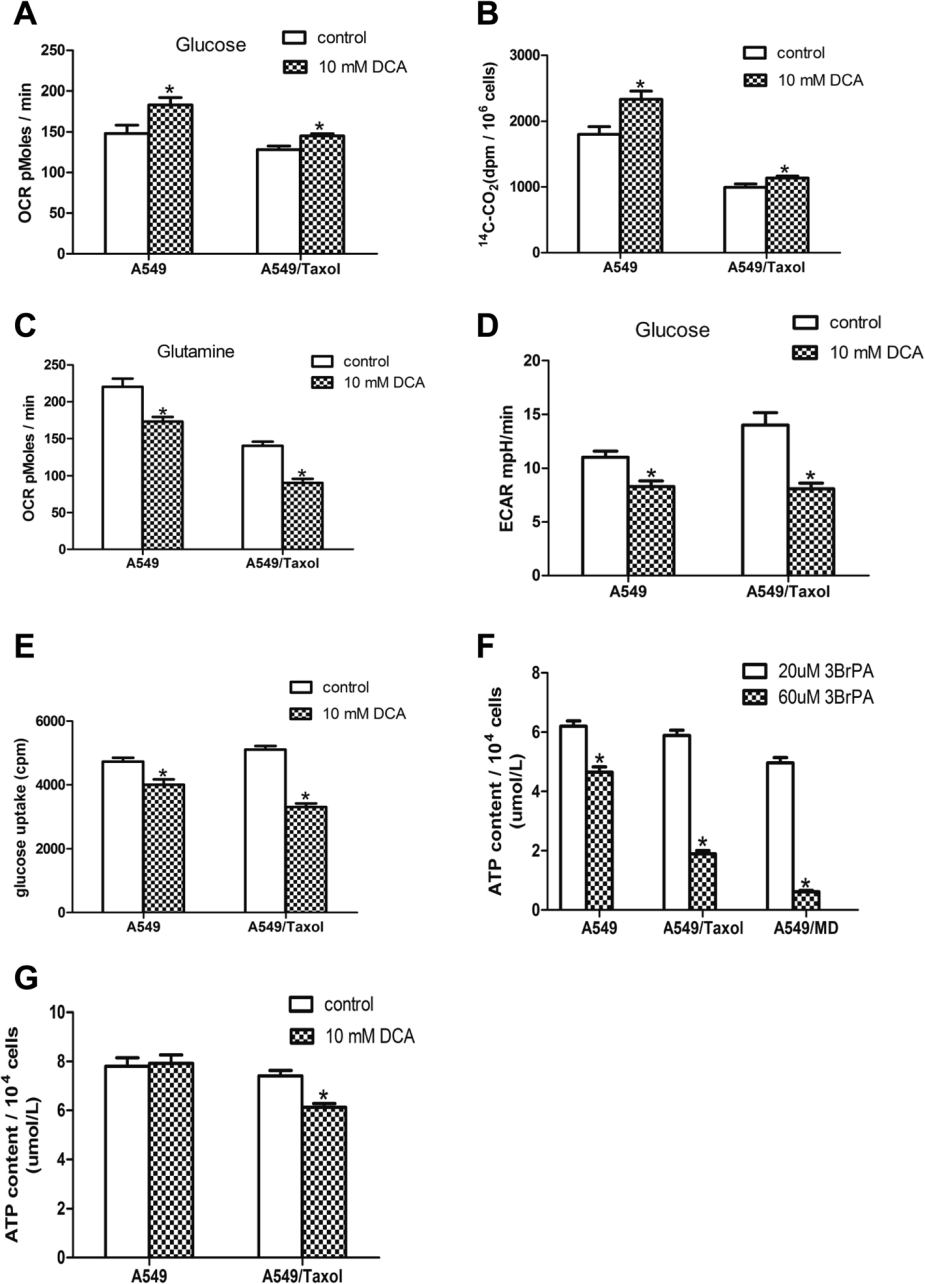
**Influence of DCA on mitochondrial respiration and glycolysis. (A)** OCR in A549 and A549/Taxol cells treated with 0 or 10 mM DCA for 24 h with glucose as carbon source. **(B)** Release of ^14^C-CO_2_ by A549 and A549/Taxol cells treated with 0 or 10 mM DCA for 24 h. **(C)** OCR in A549 and A549/Taxol cells treated with 0 or 10 mM DCA for 24 h with glutamine as carbon source. **(D)** Influence of DCA on ECAR in A549 and A549/Taxol cells. **(E)** Influence of DCA on glucose uptake in A549 and A549/Taxol cells incubated in DMEM with 18 F-FDG for 1 h. Uptake of radioactivity was measured with a well gamma counter. **(F)** Effect of 3BrPA on ATP generation by glycolysis in A549, A549/Taxol and A549/MD cells. **(G)** Influence of DCA on ATP production after treatment with 0 or 10 mM DCA for 24 h. *P < 0.05. Data are mean ± SEM of three independent experiments.

Next we assessed whether DCA specifically targets drug-resistant cells with mitochondrial respiratory defect by assisting the recovery of mitochondrial function. Glutamine and glucose are important carbon sources for oxidative phosphorylation in tumor cells [29]. DCA increased oxygen consumption by 19% in A549 cells but only by 9% in A549/Taxol cells, with glucose as a carbon source (Figure 3A). This observation was consistent with ^14^C-CO_2_ expiration data (Figure 3B). With glutamine as a carbon source, DCA significantly inhibited glutamine oxidation via a mechanism that is not understood. DCA inhibited glutamine oxidation by 34.4% in A549/Taxol cells and 19.1% in A549 cells. DCA inhibited glutamine oxidation more effectively in drug-resistant cells exhibiting mitochondrial respiratory defects (Figure 3C). These results suggest that the ability of DCA to target specifically tumor cells with mitochondrial respiratory defects is not due to the restoration of mitochondrial function in A549/Taxol cells.

Although DCA could not effectively restore mitochondrial respiration in resistant cells, DCA inhibited ECAR significantly compared to control treatment in both A549 and A549/Taxol cells. However, there was a larger difference in ECAR rate between control and DCA treated cells in the A549/Taxol cells than the A549 (Figure 3D). DCA reduced glucose uptake by 35% in A549/Taxol cells, but only by 12% in A549 cells (Figure 3E). These results were significant as glycolysis was the main source of ATP in cells with severely injured mitochondria [25]. This result was consistent with specific inhibition of glycolytic ATP generation using 3-bromopyruvate (3BrPA), which is an inhibitor of hexokinase II with an inhibitory effect on glycolysis (Figure 3F). Whether DCA decreased ATP production via inhibition of glycolysis was investigated, and 10 mM DCA for 24 h decreased ATP generation in A549/Taxol cells, but ATP production was unaffected in A549 cells (Figure 3G).

### DCA suppresses P-glycoprotein function in paclitaxel-resistant cells

**Figure 4:**
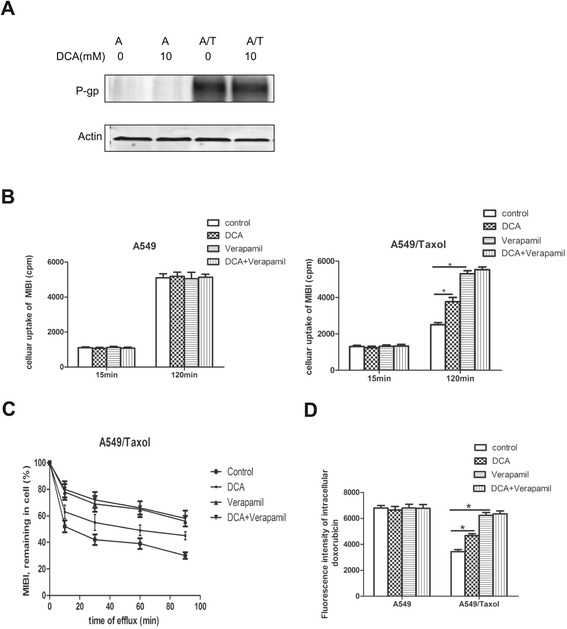
**DCA partially inactivates P-glycoprotein activity in drug-resistant cell lines. (A)** P-glycoprotein expression in A549 and A549/Taxol cells treated with 0 or 10 mM DCA for 24 h, as determined by western blotting. **(B)** Influence of DCA, verapamil, or DCA + verapamil on ^99m^Tc-MIBI uptake. Verapamil was used as a positive control. **(C)** Influence of DCA, verapamil, or DCA + verapamilon ^99m^Tc-MIBI excretion. **(D)** Influence of DCA, verapamil, or DCA + verapamilon accumulation of doxorubicin. *P < 0.05. Data are mean ± SEM of three independent experiments.

Compared with A549 cells, A549/Taxol cells expressed higher levels of P-glycoprotein, which would be expected to increase the excretion of antitumor drugs via active transport. However, this process requires an energy supply. Although DCA had no effect on P-glycoprotein expression (Figure 4A), decreased levels of ATP in DCA-treated drug-resistant cells could lead to functional inactivation of P-glycoprotein as seen in Figure 3G, and this was investigated by measuring the uptake and excretion of the P-glycoprotein radioactive substrate ^99m^Tc-MIBI [38]. Verapamil was used as a positive control, because it is a P-glycoprotein substrate that inhibits the transport of drugs by this protein. DCA treatment increased ^99m^Tc-MIBI accumulation in A549/Taxol cells by 20%, but as the parental A549 cell line did not express P-glycoprotein, DCA did not affect ^99m^Tc-MIBI uptake (Figure 4B). DCA significantly prolonged the intracellular retention of ^99m^Tc-MIBI (Figure 4C).

Doxorubicin is another known substrate of P-glycoprotein [33], and DCA treatment also significantly increased doxorubicin accumulation in A549/Taxol cells but not in A549 cells (Figure 4D). These results suggest that DCA is able to inhibit the function of P-glycoprotein in cells with mitochondrial respiratory defects.

### DCA increases citrate accumulation in cells with mitochondrial injury

**Figure 5:**
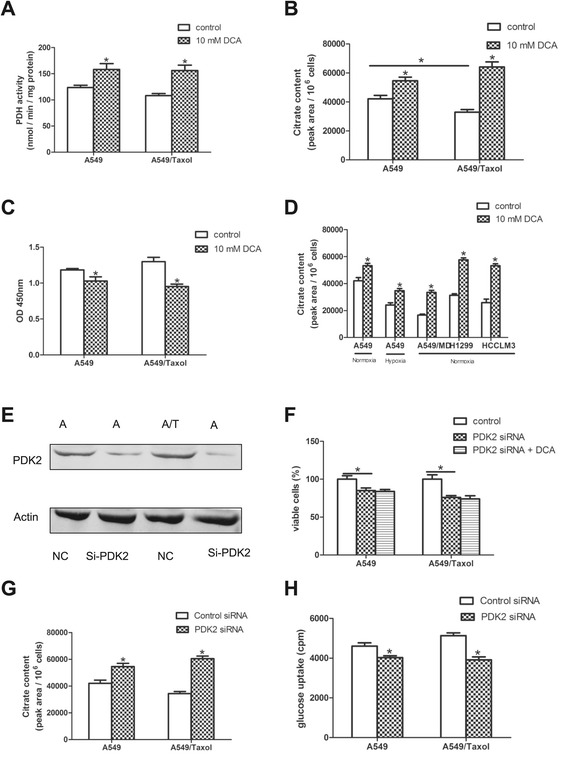
**DCA increases citrate accumulation in cells with mitochondrial injury. (A)** Effects of a 3-h DCA treatment on PDH activity in A549 and A549/Taxol cells. **(B)** Influence of DCA on citrate accumulation measured by GC. **(C)** Influence of DCA on PFK activity by PFK colorimetric assay. **(D)** Intracellular citrate levels under normoxia in various cell lines and hypoxic conditions in A549 cell lines treated with 0 or 10 mM DCA for 24 h. **(E)** Comparative analysis of PDK2 expression in control and PDK2-siRNA cell lines determined by western blotting (48 h post-transfection). **(F)** Viability of A549 and A549/Taxol cells treated with PDK2 siRNA. **(G)** Influence of PDK2 siRNA on citrate accumulation. **(H)** Glucose uptake in control and PDK2-siRNA cells. *P < 0.05. Data are mean ± SEM of three independent experiments.

The above results indicated that DCA could inactivate P-glycoprotein to enhance sensitivity of A549/Taxol cells to paclitaxel by inhibiting glycolysis. We elucidated why DCA inhibited glycolysis in A549/Taxol cells but did not activate the TCA cycle. DCA targets PDK and increases PDH activity, thus it would be expected to stimulate the TCA cycle. DCA was found to activate PDC in both A549 and A549/Taxol cells (Figure 5A), but the TCA cycle was not stimulated in either cell type. We therefore investigated whether this resulted in accumulation of TCA cycle substrates in A549/Taxol cells.

Acetyl-CoA is the product of PDH and an important intermediate in energy generation and fatty acid synthesis. However, acetyl-CoA does not pass through the mitochondrial membrane, and is quickly converted into citric acid by citrate synthase (which also has a high affinity for acetyl-CoA). Citrate is then transported out of the mitochondria via the citric acid shuttle, or enters the TCA cycle [39]. Cells harboring mitochondrial respiratory defects do not possess a normally functioning TCA cycle, therefore citric acid generated in DCA-treated cells cannot be consumed in a timely fashion, and instead accumulates in the cytoplasm. Citrate is an important metabolite that regulates the balance between glycolysis and oxidative phosphorylation in cancer cells. In the cytoplasm, citrate can specifically inhibit PFK1, an enzyme critical to glucose metabolism, to reduce glycolysis [40].

We confirmed that citrate levels induced by DCA were significantly different in A549 and A549/Taxol cells (Figure 5B). Citrate was lower in A549/Taxol cells in the absence of DCA; however,10 mM DCA treatment for 24 h resulted in an 80% increase in citrate in A549/Taxol cells, compared with a 25% increase in A549 cells (Figure 5B). Citrate accumulation resulted in inhibition of PFK activity (Figure 5C). We examined the effect of DCA on citrate generation under different conditions of mitochondrial damage, and citrate levels declined significantly in hypoxia or A549/MD cell models with mitochondrial respiratory deficiency (Figure 5D). However, citrate levels were higher after DCA treatment compared withA549 cells under normoxia. Other cell lines such as H1299 (p53 deficiency) and HCCLM3 (highly aggressive) [23] can tolerate much higher concentrations of paclitaxel compared withA549 cells, with IC50 concentrations 2.3- and 3.0-fold higher than A549 cells, respectively. Similarly, we observed that citrate levels declined and DCA treatment induced greater citrate accumulation in these cell lines compared with A549 cells.

We next used PDK2 siRNA to inhibit PDK and mimic DCA treatment. PDK2 was chosen because it is the only widely expressed isoenzyme and has the lowest Ki for DCA [41]. Treatment with PDK2 siRNA inhibited PDK2 expression in A549 and A549/Taxol cells (Figure 5E). Consistent with DCA treatment, knockdown of PDK2 for 72 h resulted in greater cell death in A549/Taxol cells than in A549 cells. However, PDK2 siRNA cells were not sensitive to DCA treatment (Figure 5 F). We confirmed that PDK2 siRNA increased citrate and decreased glucose uptake more significantly in A549/Taxol cells, which was consistent with DCA treatment (Figure 5G, H).

### DCA improved *in vivo* efficacy of paclitaxel in A549/Taxol cell xenografts

**Figure 6:**
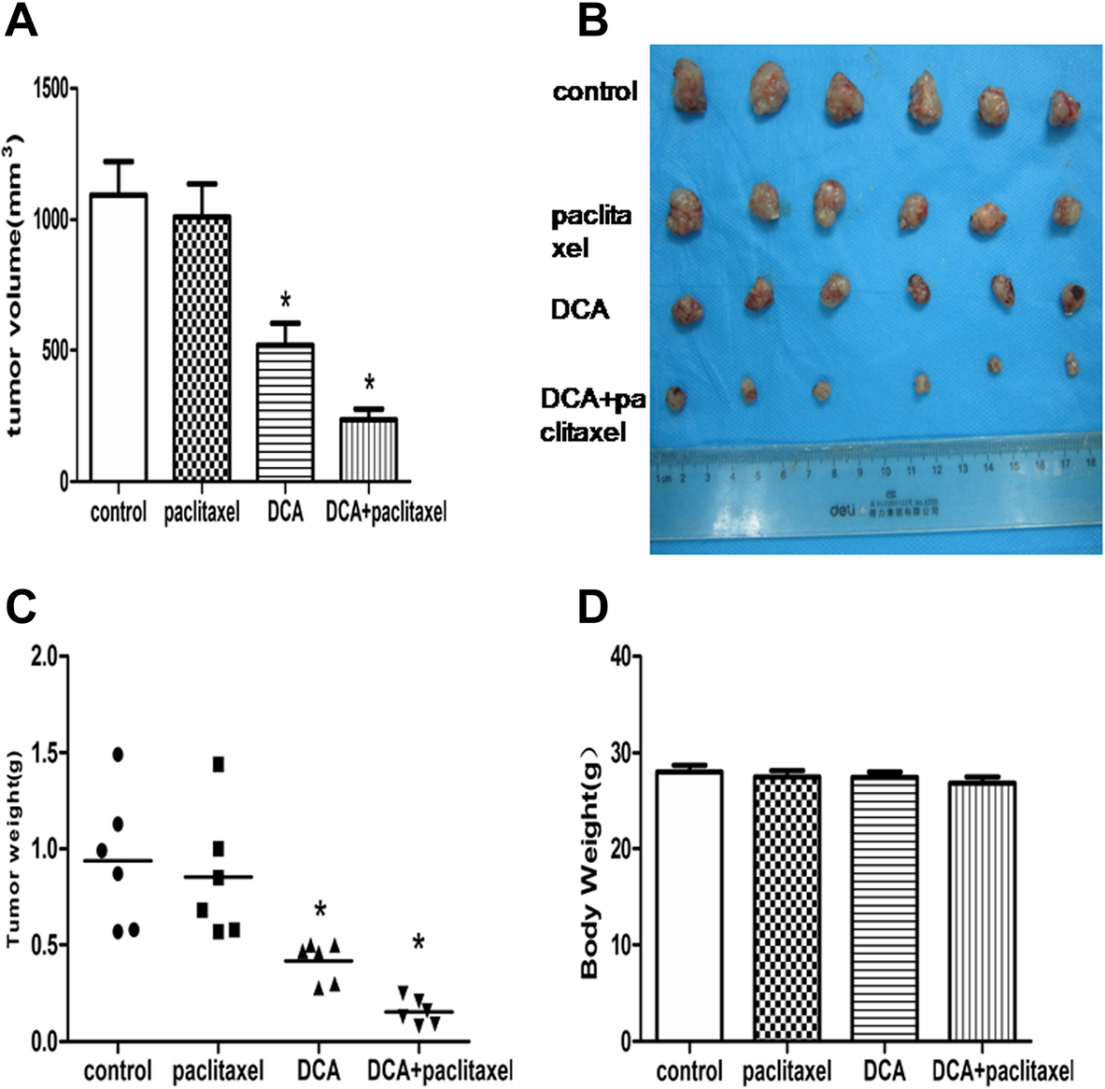
**Effect of paclitaxel and DCA alone and in combination on the growth of A549/Taxol xenografts in nude mice. (A-C)** *In vivo* growth of tumors in mice treated with DCA alone or in combination with paclitaxel was significantly inhibitedcompared with control mice, whereas treatment with paclitaxel alone had no effect. **(D)** Effect of vehicle, paclitaxel, DCA, or combined treatment on body weight. *P < 0.05. Data are mean ± SEM of three independent experiments.

Treatment with paclitaxel alone did not significantly suppress tumor volume (Figure 6A) or weight (Figure 6B) compared with the control group. In contrast, a combination of DCA and paclitaxel decreased tumor volume by 78%, compared with a decrease of only 8% with paclitaxel alone (relative tumor size to vehicle-treated tumors after 3 weeks’treatment; *P* < 0.001; Figure 6A). Similarly, combined treatment decreased tumor weight by 84% compared with9% with paclitaxel alone (relative tumor weight to vehicle-treated tumors after 3 weeks’treatment; *P* < 0.001; Figure 6B, C). No apparent body weight loss or toxicity was observed in any treatment group (Figure 6D). These results provide *in vivo* evidence that DCA restores drug sensitivity in A549/Taxol cells.

## Discussion

In this study, drug resistance to paclitaxel in tumor cells was closely linked with mitochondrial damage, and mitochondrial dysfunction persisted in A549 cells with acquired resistance. A549/MD cells with stable mitochondrial respiratory deficiency exhibited similar paclitaxel resistance. The mechanism by which mitochondrial respiratory defects cause resistance is complicated. Hypoxia-inducible factor 1 can lead to drug resistance through increased glycolysis and down-regulation of Bid and Bax. The damage of electron transport chain complexes could decrease mitochondrial apoptosis response leading to apoptosis resistance [42]. The present study confirmed that P-glycoprotein expression was significantly increased and induced injury to the electron transport chain in A549 cells. P-glycoprotein is an important resistance protein that can prevent apoptosis by excreting paclitaxel [38]. Increased P-glycoprotein expression due to mitochondrial damage is therefore a potential explanation for paclitaxel resistance in lung cancer.

In this study, DCA targeted A549/Taxol cells specifically and reversed paclitaxel resistance. Surprisingly, what sets this study apart from others is how DCA targets cells with mitochondrial respiratory defects, which was not due to its ability to activate oxidative respiration. Rather, DCA inhibited glutamine oxidation significantly between control and DCA treated cells in both cell lines. However, DCA inhibited glutamine oxidation by 34.4% in A549/Taxol cells and 19.1% in A549 cells.A549/Taxol cells were affected by DCA’s inhibition of glutamine oxidation significantly more than A549 cells (Figure 3C).

Although DCA did not activate oxidative respiration in A549/Taxol cells compared withA549 cells, and inhibited glucose uptake in both cell types, it inhibited glycolysis more effectively in A549/Taxol cells. We did observe DCA reversed paclitaxel resistance by inhibiting glycolysis. Tumor cells rely on ATP to maintain drug resistance, and decreased ATP can lead to decreased drug resistance [43]. DCA clearly decreased ATP generation in A549/Taxol cells, presumably by inhibiting glycolysis, but failed to reduce ATP production in A549 cells that exhibited better mitochondrial function.

Intracellular ATP is mainly produced by glycolysis and oxidative phosphorylation, and analysis of the main cellular energy sources showed that cells with mitochondrial respiratory defects are more dependent on energy from glycolysis. DCA significantly inhibited glycolysis and oxidative phosphorylation of glutamine in A549/Taxol cells, and increased oxidative phosphorylation of glucose could not compensate for the lost energy. This would inevitably lead to reduced ATP generation in A549/Taxol cells. P-glycoprotein is important in paclitaxel resistance, and maintenance of P-glycoprotein activity requires a steady supply of ATP [44]. Therefore, DCA may suppress P-glycoprotein activity by inhibiting glycolysis, which could restore paclitaxel susceptibility in resistant cells with mitochondrial respiratory defects.

Citric acid is generated by citrate synthase following DCA activation of PDC [29], and citrate can then enter the TCA cycle, be oxidized in mitochondria, or enter the cytoplasm via dedicated carrier proteins [39]. However, we found that the TCA cycle was not activated in cells with mitochondrial respiratory defects, suggesting accumulation of intermediates from the generation of acetyl-CoA (by activated PDC). Citrate is both a substrate of and intermediate in the TCA cycle, therefore DCA may induce the accumulation of citrate in cells with mitochondrial respiratory defects. In this study, DCA induced greater levels of citrate accumulation (80%) in resistant A549/Taxol cells than in sensitive A549 cells. Similarly, DCA induced greater accumulation of citrate in other cell types with mitochondrial respiratory defects, including A549/MD cells that are deficient in mitochondrial respiration under hypoxic conditions.

Citrate lies in a central position in the regulation of cell metabolism and participates in the synthesis of fatty acids in the cytoplasm. Citrate inhibits PFK1 to reduce glycolysis and operates as a key regulator, balancing oxidation and glycolysis [39]. We noted that cells with mitochondrial respiratory defects had decreased levels of citrate, which is conducive for maintaining the Warburg effect to balance energy supply and biosynthesis. Citrate may therefore be used as an indicator of mitochondrial respiratory defects and paclitaxel resistance in tumors, and future metabolic studies on tumors should take this into account.

## Conclusions

In summary, paclitaxel-resistant cells exhibited increased mitochondrial respiratory defects and decreased citric acid levels. DCA induced citrate accumulation that inhibited glycolysis and led to inactivation of P-glycoprotein and reduced drug resistance. These results will be useful for future treatment using DCA alone or in combination with other chemotherapeutic drugs, and aid our understanding of the metabolic regulatory mechanisms in tumors.

## Additional files

Additional file 1: Figure S1. A549/MD cells have minimal complex I activity than A549 cells. *indicate significant differences (p <0.05). Data are means ± SEM of three independent experiments. Additional file 2: Figure S2. Expression of P-gp was increased in A549/MD cells compared to A549 cells. *indicate significant differences (p <0.05). Data are means ± SEM of three independent experiments.

## Abbreviations

3BrPA: 3-bromopyruvate
DCA: Dichloroacetate
DMEM: Dulbecco’s modified eagle’s medium
ECAR: Extracellular acidification rate
FDG: Fluorodeoxyglucose
MIBI: Methoxyisobutylisonitrile
OCR: Oxygen consumption rate
PDC: Pyruvate dehydrogenase complex
PDH: Pyruvate dehydrogenase
PDK: Pyruvate dehydrogenase kinase
PFK1: Phosphofructokinase1
TCA cycle: Tricarboxylic acid cycle
