# Supplementary material for: Dichloroacetate restores drug sensitivity in paclitaxel-resistant cells by inducing citric acid accumulation
Source: Mol Cancer. 2015 Mar 19;14:63. doi: 10.1186/s12943-015-0331-3 (PMC4379549; doi:10.1186/s12943-015-0331-3)

Figure S1.

A549/MD cells have minimal complex I activity than A549 cells. * indicate significant differences (p <0.05). Data are means ± SEM of three independent experiments.


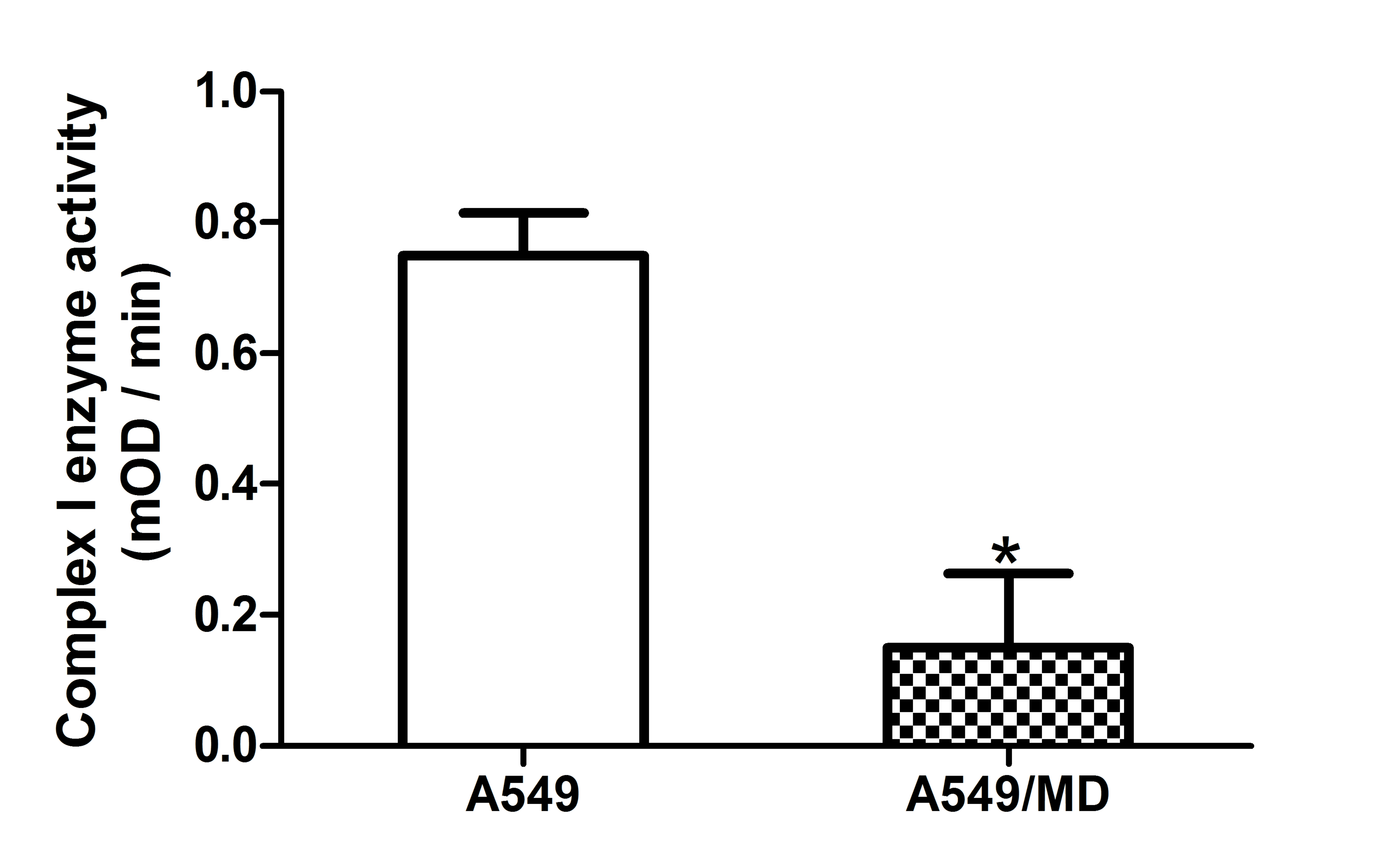

Supplement: Additional file 1: Figure S1. — A549/MD cells have minimal complex I activity than A549 cells. *indicate significant differences (p <0.05). Data are means ± SEM of three independent experiments. [file 12943_2015_331_MOESM1_ESM.docx]
