# Supplementary material for: Dichloroacetate restores drug sensitivity in paclitaxel-resistant cells by inducing citric acid accumulation
Source: Mol Cancer. 2015 Mar 19;14:63. doi: 10.1186/s12943-015-0331-3 (PMC4379549; doi:10.1186/s12943-015-0331-3)
Supplement: Additional file 2: Figure S2. — Expression of P-gp was increased in A549/MD cells compared to A549 cells. *indicate significant differences (p <0.05). Data are means ± SEM of three independent experiments. [file 12943_2015_331_MOESM2_ESM.docx]

Figure S2.

Expression of P-gp was increased in A549/MD cells compared to A549 cells. * indicate significant differences (p <0.05). Data are means ± SEM of three independent experiments.


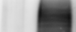

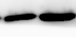


**P-gp**

**Actin**

**A549**

**A549/MD**
